# Future without delirium: not quite there yet but we can start by prescribing touch

**DOI:** 10.1186/s13054-022-04147-1

**Published:** 2022-10-27

**Authors:** Susana M. Fernandes, Maria Adão-Serrano, Ana Rita Rodrigues, Patrícia Belo

**Affiliations:** 1 Faculdade de Medicina da Universidade de Lisboa, Clínica Universitária de Medicina Intensiva, Avenida Professor Egas Moniz, 1649-028 Lisbon, Portugal; 2 Unidade de Urgência Médica, Hospital de São José, Centro Hospitalar Universitário Lisboa Central, Lisbon, Portugal; 3 Serviço de Medicina Intensiva, Hospital de Santa Maria, Centro Hospitalar Universitário Lisboa Norte, Lisbon, Portugal; 4 Grupo de Investigação e Desenvolvimento em Infeção e Sepsis, Porto, Portugal

The recently published article by Kotfis et al. “The future of intensive care: delirium should no longer be an issue” [1], brought us great interest and although we agree with the authors' perspective this is still far from being reality.

Despite delirium increase report as an adverse event, it is often faced on daily practice as a patient's “weakness” or an inevitability due to acute illness that we fail to avoid. This perspective precludes multidisciplinary team effort from targeting the modifiable components of delirium mentioned in the paper.

The COVID-19 pandemic and patients’ isolation in the ICUs have changed us; we struggle to implement concepts that might bring us closer to a delirium free future. Nevertheless, the constraints are still deeply buried in the critical care culture.

It is in the intensive care units (ICU) where individuals are most deprived from humanity. Like in Monthy Python’s 1983 film “The meaning of life”, monitors that “beep”, catheters and tubes often lead critical care professionals to forget the humanity in the patient. In our ICUs, clinicians spend little time with patients, occupied with data coming from monitors, ventilators, and other tests; many times, these replace physical examination and an opportunity to reach the human is lost. Nurses underpaid and overwhelmed with paperwork and drug preparation, often struggle reaching out to the patient, wishing for a quiet shift. Here lies some of the constraints to the “G” letter in the proposed A-I bundle.

In addition, we are quick to ask for monitoring devices and technology, in detriment of changes that may increase patient comfort, encourage mobilization, and contribute to decreasing delirium. And so, as new ICUs in our country are being equipped with the latest flagship medical technology, sunlight, noise control and adequate family spaces (amongst others), are disregarded or viewed as a non-affordable luxury.

In addition, during the process of waking a patient, we often still hear: “do not untie them, they’ll remove the tube”, “they’re agitated, please give them drugs”, “they cannot sleep, please give them drugs” and so on. Often as patients awaken and hands are untied, we realize they just want to scratch their noses, move their heads away from bright light, avoid painful stimuli or simply search for the sunlight reminding them they are alive.

We believe that just like we handover vasopressor dose or ventilator support, we should also focus on our patients' stories and needs. When patients' reactions are often of fear we may act as enlightening and appeasing elements. Show them that they're not alone and that we know who they are. Nevertheless, we feel that the non-pharmacologic approach to delirium is relegated to a secondary role.

We remember times during the COVID-19 pandemic, when wide-eyed patients were certain that we wanted to kill them or that someone was in the room waiting to cause harm. After weeks of deep sedation their first words were often “Hide, they are out there to kill us. Can’t you see?”. As we stayed by their side reminding them where they were, many in the team considered our efforts a waste of time. Sedatives were frequently a quicker solution when human resources were scarce.

We often concluded that increasing respiratory support or drugs did not help much [2], but things like a children's game of word search could change everything. Simple gestures, playing heavy metal music, buying chocolate, a homemade dessert on the tip of the tongue, a hand massage or a few comforting words helped ease their minds. Each patient is different, but time by their side is needed to get to know more about who they are.

During the pandemic, staffing was scarce, hospitals restricted family visits and accordingly delirium incidence shot up [3]. We set up a medical student volunteering scheme in collaboration between the medical school and the critical care department. Students spent time by the bedside comforting and reorienting patients. “Do not be afraid” was sometimes enough to decrease their heart and breathing rates, allowing for a decrease in sedation.

As this work was implemented, “Wake-up Nanda” became a symbol of last years’ struggle in persuading peers to embrace this proximity approach. Of all the work we have done in the ICU, this was the most rewarding.

Although this message has often been repeated in the last years [2], it is far from universally implemented. No trial has yet proven that human touch, or sunlight in the ICU improves prognosis [4], but is this not common sense? If improving the patient's environment and promoting humanization of care is not yet seen as a top priority, we hope that at least prolonged family visits become a paradigm. And at the end maybe we should start with a very simple change: prescribing human touch.

## Data Availability

Not applicable.
