# Supplementary material for: Future without delirium: not quite there yet but we can start by prescribing touch
Source: Crit Care. 2022 Oct 27;26:332. doi: 10.1186/s13054-022-04147-1 (PMC9610330; doi:10.1186/s13054-022-04147-1)
Supplement: Related texts

# Reply: Authors’ response

**Authors:** Katarzyna Kotfis, Irene van Diem-Zaal, Shawniqua Williams Roberson, Marek Sietnicki, Mark van den Boogaard, Yahya Shehabi, E. Wesley Ely

**Affiliations:** 5 grid.107950.a 0000 0001 1411 4349 Department of Anesthesiology, Intensive Therapy and Acute Intoxications, Pomeranian Medical University in Szczecin, Szczecin, Poland; 6 grid.10417.33 0000 0004 0444 9382 Department of Intensive Care, Radboud Institute for Health Sciences, Radboud University Medical Center, Nijmegen, The Netherlands; 7 grid.7692.a 0000000090126352 Department of Intensive Care Medicine, University Medical Center Utrecht, Utrecht, The Netherlands; 8 Critical Illness, Brain Dysfunction, and Survivorship (CIBS) Center, Center for Health Services Research, Nashville, TN USA; 9 grid.412807.8 0000 0004 1936 9916 Department of Neurology, Vanderbilt University Medical Center, Nashville, TN USA; 10 grid.152326.1 0000 0001 2264 7217 Department of Biomedical Engineering, Vanderbilt University, Nashville, TN USA; 11 grid.411391.f 0000 0001 0659 0011 Department of Architecture, West Pomeranian University of Technology in Szczecin, Szczecin, Poland; 12 grid.1002.3 0000 0004 1936 7857 Monash Health School of Clinical Sciences, Monash University, Melbourne, VIC Australia; 13 grid.1005.4 0000 0004 4902 0432 School of Clinical Medicine, University of New South Wales, Sydney, NSW Australia; 14 grid.412807.8 0000 0004 1936 9916 Division of Allergy, Department of Medicine, Pulmonary, and Critical Care Medicine, Vanderbilt University Medical Center, Nashville, TN USA; 15 Geriatric Research, Education and Clinical Center (GRECC) Service, Nashville Veterans Affairs Medical Center, Tennessee Valley Healthcare System, Nashville, TN USA

We would like to thank Fernandes and colleagues for raising several important issues relating to our Viewpoint published in Critical Care [1]. We fully agree that ICU design and organization deprive patients of humanity. The COVID-19 pandemic has caused a major global decrease in the quality of care, but despite its overwhelming burden, it could help us re-design the way we think about ICU.

Humanizing care is a general attitude of professionals toward patients and relatives to provide holistic care [5]. We fully agree that bringing sunlight, fresh air and sound control are common sense interventions that cannot be regarded as luxury, but normality. To humanize ICUs, we all need empathic clinician-patient encounter. Basile et al. have shown that negative behaviors of ICU clinicians leave patients and families dehumanized; this may delay patient recovery, bring mental illness, and cause lack of trust [6]. The idea of ICU liberation was designed to implement evidence-based pain, agitation, and delirium guidelines by using the ABCDEF bundle to humanize ICU care [7]. We now extend it towards gaining insight and redesigning ICUs to restore a sense of human dignity.

We agree that starting by “*prescribing touch”* is an easy and accessible intervention that carries no financial cost. As the authors rightly mention quality of care is a measure of humanity in the ICU brought by simple gestures and finding a person in the patient in front of us. Although no trial has shown the efficacy of human touch in the adult ICU, it has been proven to be a standard of care in neonatal ICUs, with advanced differentiation between ‘procedural touch’ and ‘comforting touch stimulation’ [8]. Maybe it is time to introduce these terms into adult ICU.

Technology advances are integral components of the ICU but must be balanced with caring for the patient as an autonomous person. We must teach students and remind ourselves that fine monitoring and high technology equipment cannot replace a focused physical examination and a thinking, caring professional. It is also easy to find an excuse for losing humanity, such as underpayment or paper overload, but changing working conditions should involve the hospital ethical boards and management teams. We agree that the changes take time and, in many cases, both healthcare teams and hospital management may be reluctant to modify the ICU culture, but we believe that the changes are inevitable and will be requested by highly aware families.
